# UNDERSTANDING LEADERSHIP IN NURSING HOME CONTEXT THROUGH ADAPTIVE LEADERSHIP FRAMEWORK FOR CHRONIC ILLNESS

**DOI:** 10.1093/geroni/igac059.1238

**Published:** 2022-12-20

**Authors:** Jing Wang, Janelle Perez, Anna Beeber, Ruth Anderson, Whitney Berta, Holly Lanham, Carole Estabrooks

**Affiliations:** University of North Carolina at Chapel Hill, Chapel Hill, North Carolina, United States; University of North Carolina Chapel Hill, Chapel Hill, North Carolina, United States; Johns Hopkins University, Baltimore, Maryland, United States; UNC Chapel Hill, Chapel Hill, North Carolina, United States; University of Toronto, Toronto, Ontario, Canada; University of Texas Health San Antonio, San Antonio, Texas, United States; University of Alberta, Edmonton, Alberta, Canada

## Abstract

The case studies were conducted as an early component of a pan-Canada project entitled Translating Research in Elder Care (TREC). This sub-project provided insights into the challenges and leadership of facilitating care model changes, care quality improvement, and quality of work enhancement of three long-term care (LTC) facilities in Canada. Through the lens of Adaptive Leadership Framework for Chronic Illness (ALFCI), leadership emerged as a critical element of their organizational context. Staff reported that contextual factors of high intensity of work and inadequate staff were barriers that added to the complexity of challenges facing them. Data collectors observed that frontline staff exhibited leadership behaviors in knowledge transmission, information sharing, teamwork, and person-centered strategies to address challenges. However, top-down facilitation can lead to misunderstanding and a lack of motivation from the frontline staff to follow the facilitation. The findings also suggested tailored facilitation about including frontline staff in formal interactions.

